# SIRT2 is involved in the modulation of depressive behaviors

**DOI:** 10.1038/srep08415

**Published:** 2015-02-12

**Authors:** Rui Liu, Wei Dang, Ying Du, Qiong Zhou, Kai Jiao, Zhaohui Liu

**Affiliations:** 1 Department of Rehabilitation, Tangdu Hospital, Fourth Military Medical University, Xi'an 710032, P.R. China; 2 Department of Psychiatry, Xi'an Mental Health Center, Xi'an 710061, P.R. China; 3 Department of Neurology, Tangdu Hospital, Fourth Military Medical University, Xi'an 710032, P.R. China; 4 Department of Endocrinology, Tangdu Hospital, Fourth Military Medical University, Xi'an 710032, P.R. China

## Abstract

Exposure to chronic stress produces negative effects on mood and hippocampus-dependent memory formation. SIRT2 alteration has been reported in mood disorders; however, the role of SIRT2 in depression remains unclear. Therefore, we aimed to determine whether SIRT2 can restore stress-induced suppression of neurogenesis in a rat chronic unpredictable stress (CUS) model of depression. Sucrose preference test, home-cage locomotion, forced swim test, and elevated plus maze were used to determine the role of SIRT2 in CUS model. To further determine the hippocampal neurogenesis contributes to the role of SIRT in mediating the antidepressant-like behavior, rats were exposed to X-irradiation to disrupt the process of hippocampal neurogenesis. CUS decreased expression of the SIRT2 protein in the hippocampus. Treatment with the antidepressant fluoxetine reversed the CUS-induced SIRT2 change. Furthermore, inhibiting SIRT2 by tenovin-D3 resulted in depression-like behaviors and impaired hippocampal neurogenesis in rats. Conversely, overexpression of SIRT2 by the intra-hippocampal infusion of recombinant adenovirus vector expressing mouse SIRT2 reversed the CUS-induced depressive-like behaviors, and promoted neurogenesis. Disrupting neurogenesis in the dentate gyrus by X-irradiation abolished the antidepressant-like effect of Ad-SIRT2-GFP. These findings indicate that hippocampal SIRT2 is involved in the modulation of depressant-like behaviors, possibly by regulating neurogenesis.

Depression is a common disorder worldwide and is associated with an increased risk of suicide, impaired social skills, social withdrawal and substance abuse^1^. Human depression has a heterogeneous etiology; therefore, the underlying mechanisms appear to be diverse and complex. The treatment of depression is confounded by the high rates of treatment resistance, coupled with a low probability of achieving lasting remission. Classically prescribed monoaminergic modulators regularly lead to measurable improvements in only half of the depressed clinical population, and remission in less than 30–40%^2^. Therefore, it is urgently required to identify and develop novel alternative therapeutic approaches based on validated disease mechanisms to treat depression and related mood disorders.

Sirtuins (SIRTs) are class III histone deacetylases whose activities are dependent on and regulated by nicotinamide adenine dinucleotide (NAD^+^)^3^. SIRTs modulate major biological pathways, such as stress response, protein aggregation, and inflammatory processes, which are involved in neurodegenerative diseases^4^. In mammals, there are seven sirtuins, SIRT1-7, all of which possess a highly conserved central NAD^+^-binding site and common catalytic domain. Among all mammalian SIRTs, SIRT1 has been the most extensively studied, and accumulating evidence suggests that SIRT1 plays a protective role in normal brain physiology and neurological disorders^5^. The cognitive deficits in SIRT1 knockout mice or mutant mice lacking SIRT catalytic activity are associated with defects in synaptic plasticity in the hippocampus^6^. SIRT1 knockout mice exhibit a decrease in dendritic branching, branch length and complexity of neuronal dendritic arbors, and show altered hippocampal gene expression, which plays important roles in synaptic and structural functions^7^, suggesting that SIRT1 plays an important role in neurological disorders.

Like SIRT1, SIRT2 is a strong deacetylase with some common substrates in the cytoplasm and nucleus^8^. Interestingly, a recent study reported that altered SIRT1, 2 and 6 mRNA expression in peripheral blood cells may be useful biological markers for mood disorders^9^. Despite the data indicating an association between SIRT2 and neurodegenerative disorders, there is no direct evidence that SIRT2 protein levels in the hippocampus can actually affect behaviors associated with depression.

In this study, we examined the effects of SIRT2 on hippocampal neurogenesis and behaviors in a chronic unpredictable stress model of depression and the involvement of hippocampal neurogenesis in the antidepressant-like behavioral effects of SIRT2. These results suggested that the involvement of hippocampal neurogenesis is required for the antidepressant-like behavioral effects of Ad-SIRT2. Our data led us to conclude that SIRT2 is essential for normal mouse cognitive functions.

## Results

### Implication of hippocampal SIRT2 alternations in depressive behaviors

**Figure 1:**
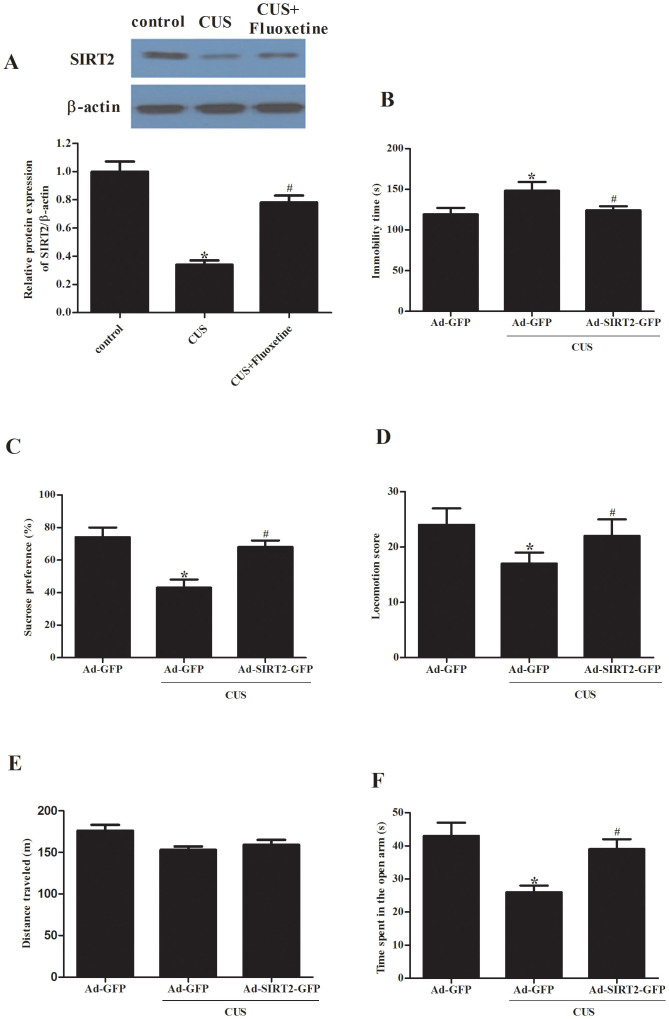
Implication of hippocampal SIRT2 alternations in depressive behaviors. (A), the rats were exposed to CUS for 49 d and treated with fluoxetine during the last 28 d of CUS, and the protein expression levels of SIRT2 in different groups were determined by western blot analysis on the next day. Ad-SIRT2-GFP or Ad-GFP was delivered into the DG of rats by microinjection; 4 d later, the rats were exposed to CUS for 21 d, and immobility time in forced swimming test (B), sucrose preference (C), location score in home-cage locomotion test (D), distance traveled (E), time spent in the open arm in the elevated plus maze test (F) were examined on the next day. Data are mean ± SD. *P < 0.05, compared with Ad-GFP-treated rats; #*P* < 0.05, as compared with Ad-GFP CUS rats.

We investigated whether CUS exposure changed the expression of SIRT2. As shown in Figure 1A, CUS exposure for 21 d led to a significant decrease in SIRT2 in the hippocampus, suggesting a correlation of chronic stress with SIRT2.

To examine whether antidepressants affected the CUS-induced SIRT2 change, we treated rats with fluoxetine, from day 22 to day 49, during 49 d CUS exposure and measured SIRT2 levels in the hippocampus. Mice exposed to CUS for 49 d exhibited a significantly decreased SIRT2 protein level, as compared with the control group; Fluoxetine treatments reversed the CUS-induced SIRT2 (Figure 1A).

To investigate whether hippocampal SIRT2 is implicated in depression-related behaviors, we constructed an overexpression SIRT2 vector. 2 μL of Ad-SIRT2-GFP or Ad-GFP was microinjected into the DG of rats. After four days, rats subjected to a CUS procedure for 21 d were assessed in the behaviors tests. As compared with the Ad-GFP group, in the forced swimming test, CUS rats microinjected with the Ad-SIRT2-GFP displayed decreased immobility time (Figure 1B). In the sucrose preference test, CUS rats microinjected with the Ad-SIRT2-GFP showed increased preference for sucrose solution (Figure 1C). In addition, home-cage locomotion score, but not exploration of a novel environment, was increased in CUS rats microinjected with the Ad-SIRT2-GFP, as compared with the Ad-GFP group (Figure 1D, E). Analysis of behavior in the elevated plus test showed lower indices of anxiety in Ad-SIRT2-GFP rats than in controls: Ad-SIRT2-GFP rats displayed more entries, spent more time in the open arms, and moved more in all areas of the elevated plus maze (Figure 1F). These results suggested that hippocampal Ad-SIRT2-GFP microinjection prevented the CUS-induced immobility time prolongation in the forced swimming test, sucrose preference reduction in the sucrose preference test and indices of anxiety decrease in the elevated plus maze; this indicated that SIRT2 was involved in CUS-induced depression behavior and SIRT2 overexpression in the hippocampus produces an antidepressant-like effect.

### Inhibiting hippocampal SIRT2 results in depressive-like phenotype

**Figure 2:**
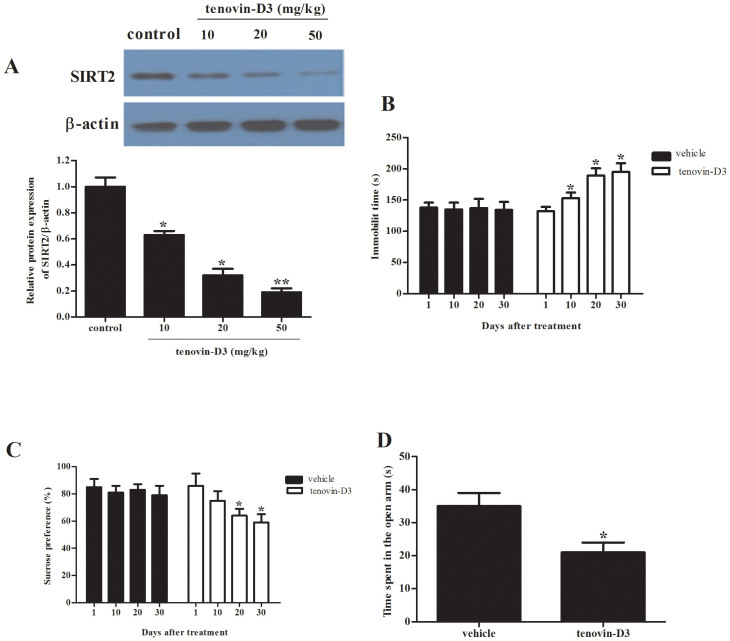
Inhibiting hippocampal SIRT2 results in depressive-like phenotype. (A), tenovin-D3 decreased the expression of SIRT2 in a dose-dependent manner; Rats were treated with tenovin-D3 for 14 d and measured immobility time in forced swimming test (B), sucrose preference (C), time spent in the open arm in the elevated plus maze test (D) at day 1, 10, 20 or 30 after withdrawal. Data are mean ± SD. *P < 0.05, compared with vehicle rats.

Since SIRT2 overexpression in the hippocampus has an antidepressant-like effect, we asked whether SIRT2 inhibition produces depression-related behaviors. To explore the effect of SIRT2 inhibition on depression-related behaviors, we treated rats with tenovin-D3 for 14 days. As shown in Figure 2A, we found that tenovin-D3 inhibited the expression of SIRT2 in the hippocampus, exhibiting a dose-dependent manner.

After treatment with tenovin-D3, the immobility time in the forced swimming test was significantly increased (Figure 2B), while the percentage sucrose preference in the sucrose preference test was significantly decreased (Figure 2C). In addition, tenovin-D3-treated rats showed fewer entries, spent less time in open arms, and moved less in all areas of elevated plus maze, as compared with vehicle (Figure 2D). These results suggested that inhibiting hippocampal SIRT2 results in depressive-like behaviors.

### Effect of SIRT2 on hippocampal neurogenesis *in* *vivo*

**Figure 3:**
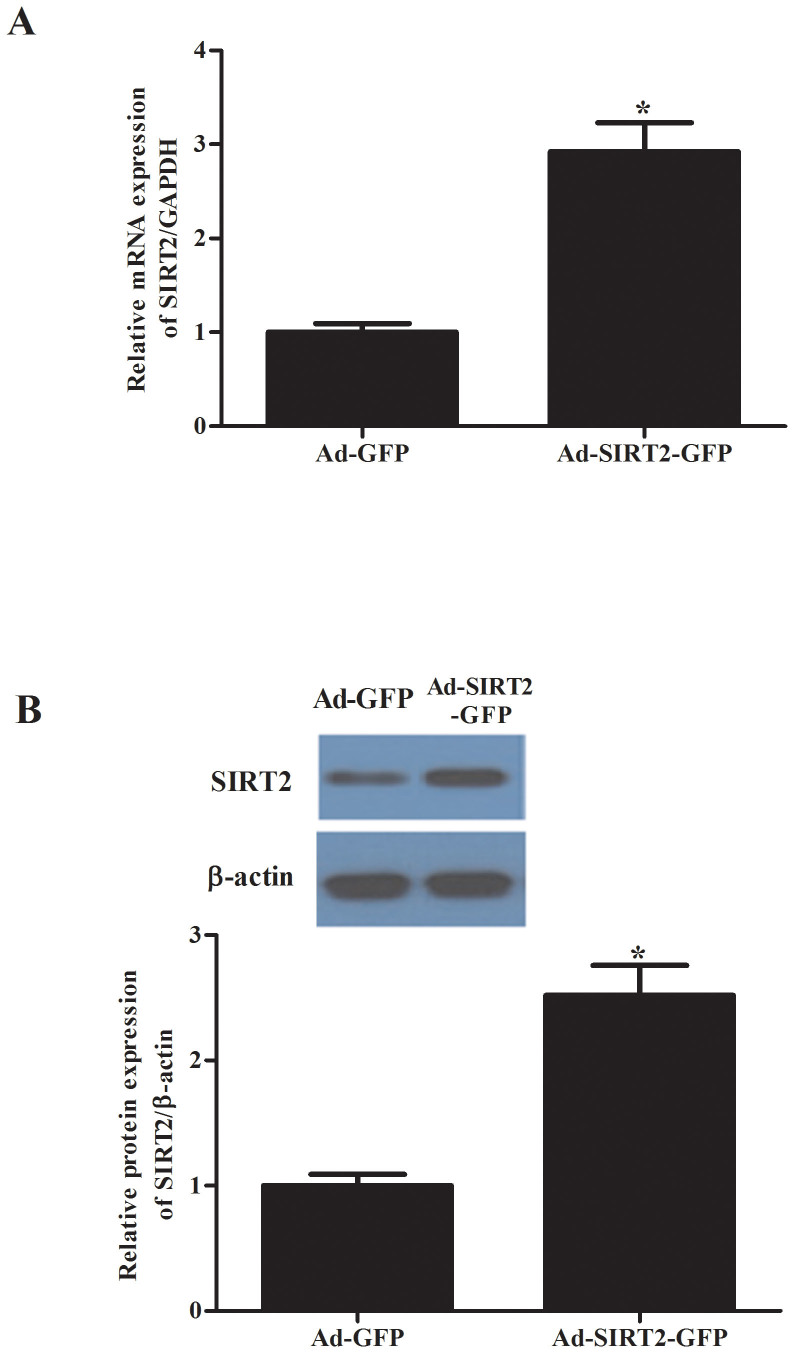
The expression of SIRT2 in hippocampus after microinjection with Ad-SIRT2-GFP and Ad-GFP. Ad-SIRT2-GFP and Ad-GFP were transfected into mouse hippocampus by stereotaxic microinjection at 1 μL per dentate gyrus. Five days later, rats were perfused transcardially, and hippocampal tissues were made. (A), SIRT2 mRNA expression in hippocampal tissue by RT-PCR analysis; (B), SIRT2 protein expression in hippocampal tissue by western blot analysis. *P < 0.05, compared with the AdGFP group.

**Figure 4:**
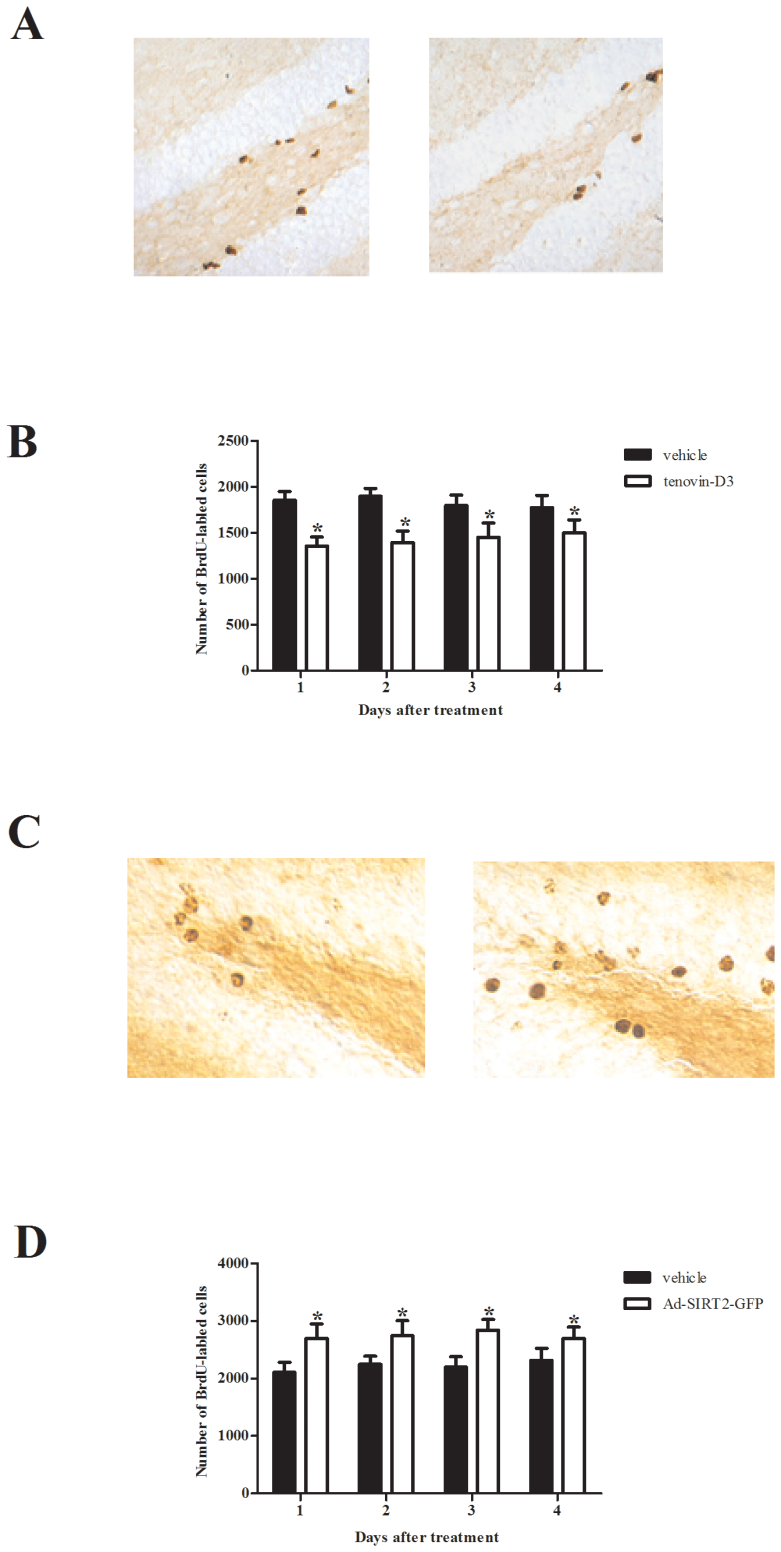
Effect of SIRT2 on hippocampal neurogenesis *in* *vivo*. (A), Representative BrdU-positive cells in the DG at 1 d after Tenovin-D3 microinjection; (B), Tenovin-D3 significantly reduced the number of BrdU-positive cells in a time-dependent manner; (C), Representative BrdU-positive cells in the DG of rats transfected with Ad-SIRT2-GFP at 28 d after BrdU labeling. (D), the dentate gyrus transfected with Ad-SIRT2-GFP markedly increased BrdU incorporation. Data are mean ± SD. *P < 0.05, compared with vehicle rats.

It has been reported that CUS that can induce depression-like behaviors could decrease the proliferation of neural cells in the dentate gyrus, which suggested that a reduction of hippocampal neurogenesis is a common feature of stress^10^. Therefore, we investigated the effect of SIRT2 on hippocampal neurogenesis *in* *vivo*. First, we investigated the expression levels of SIRT2 in hippocampus after microinjection with Ad-SIRT2-GFP and Ad-GFP. As shown in Figure 3, Ad-SIRT2 significantly increased the expression of SIRT2 in hippocampal tissue. Then, we performed a BrdU incorporation assay to identify the effect of SIRT2 inhibition on hippocampal neurogenesis. CUS rats microinjected with SIRT2 inhibitors. Tenovin-D3 significantly reduced the number of BrdU-positive cells in a time-dependent manner (Figure 4A and 4B). These results indicated that inhibiting SIRT2 could inhibit hippocampal neurogenesis.

We next examined the effect of SIRT2 overexpression on hippocampal neurogenesis. Ad-SIRT2-GFP or Ad-GFP was delivered into the hippocampus of mice by stereotaxic microinjection. As indicated in Figure 4C and 4D, compared with the Ad-GFP group, the dentate gyrus transfected with Ad-SIRT2-GFP markedly increased BrdU incorporation. Taken together, these results suggest that SIRT2 may play a vital role in hippocampal neurogenesis.

### Involvement of hippocampal neurogenesis in mediating the antidepressant-like behavioral effects of Ad-SIRT2

**Figure 5:**
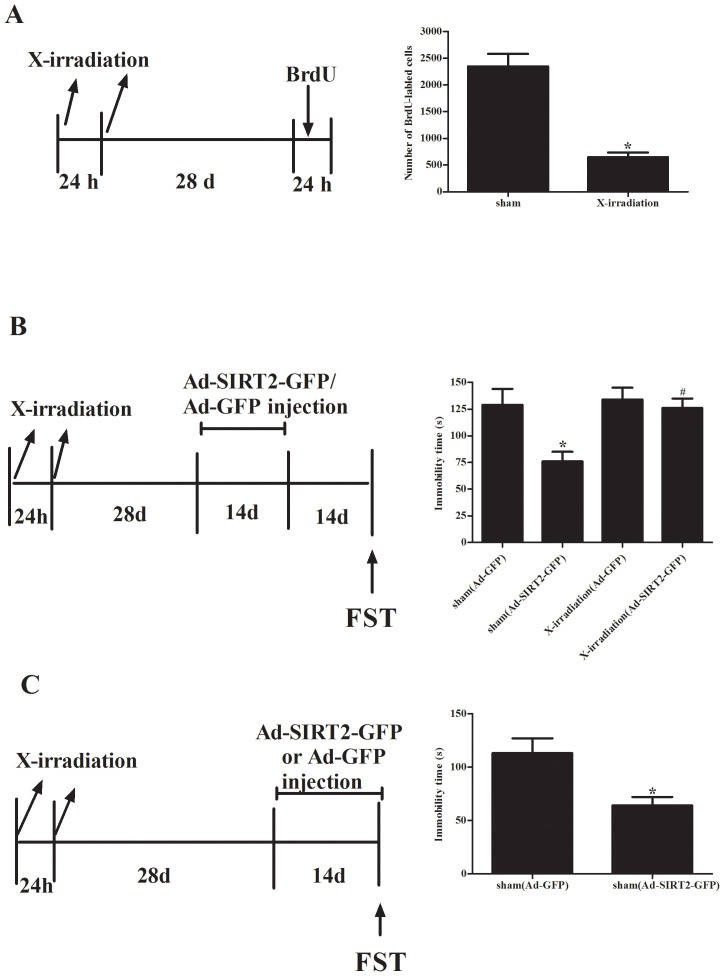
Involvement of hippocampal neurogenesis in mediating the antidepressant-like behavioral effects of Ad-SIRT2. (A), ablation of hippocampal neurogenesis by X-irradiation. The number of BrdU-labeled cells at 28 days after exposure to the sham procedure or X-irradiation, and the number of BrdU-labeled cells was decreased by X-irradiation; (B), Rats received Ad-SIRT2-GFP or Ad-GFP injection for 14 consecutive days beginning 28 days after exposure to the sham procedure or X-irradiation. The forced swim test was performed 14 days after the cessation of Ad-SIRT2-GFP treatment, and quantitative data showing the effects of X-irradiation and SIRT2 treatment on forced swim test (FST). Data are mean ± SD. *P < 0.05, compared with the sham (Ad-GFP) group, #*P* < 0.05 compared to the sham-SIRT2-GFP group. (C), antidepressant-like behavioral effects of Ad-SIRT2-GFP treatment. Rats received received Ad-SIRT2-GFP or Ad-GFP injection for 14 consecutive days beginning 28 days after exposure to the sham procedure, followed by the forced swim test. Data are mean ± SD. *P < 0.05, compared with the sham (Ad-GFP) group.

To further determine the hippocampal neurogenesis contributes to the role of Ad-SIRT2-GFP in mediating the antidepressant-like behavior, rats were first exposed to sham procedure or X-irradiation (10 Gy/day for 2 consecutive days) to disrupt the process of hippocampal neurogenesis. At 28 days after exposure to the sham procedure or X-irradiation, one set of rats was injected with BrdU to examine the effectiveness of X-irradiation in blocking neurogenesis. We found that X-irradiation greatly reduced BrdU-labeled cells in the dentate gyrus, which confirmed the ablation of hippocampal neurogenesis (Figure 5A).

To determine whether the proliferation of neurons induced by Ad-SIRT2-GFP contribute to its antidepressant-like efficacy, 28 days after X-irradiation, rats were treated for 14 days with Ad-SIRT2-GFP to up-regulate neurogenesis, followed by a 14-day delay to permit functional integration of new-born neurons during Ad-SIRT2-GFP treatment. As shown in Figure 5B, Ad-SIRT2-GFP-reduced immobility time was reversed in X-irradiation-treated rats. In addition, Ad-SIRT2-GFP treatment significantly increased swimming time, whereas the X-irradiation abolished the effect of Ad-SIRT2-GFP on swimming. To determine the immediate effects on immobility and swimming in the forced swim test following Ad-SIRT2-GFP treatment, rats exposed to the sham procedure were tested at the end of 14-days of Ad-SIRT2-GFP treatment. Ad-SIRT2-GFP rats showed a significant decrease in immobility time and an increase in swimming time (Figure 5C). These results suggest that Ad-SIRT2-GFP produces rapid and delayed antidepressant-like behavioral effects, and these effects are likely mediated, at least in part, by neurogenesis-dependent mechanisms.

## Discussion

Stress has been demonstrated to induce the suppression of hippocampal neurogenesis and behavioral phenotypes mimicking those seen in depression, which can by reversed by the administration of antidepressants^11^. In this study, we have identified the expression of SIRT2 in the hippocampus; treatment with Ad-SIRT2-GFP reversed the CUS-induced a reduction of depression-like behaviors and hippocampal neurogenesis. Ad-SIRT2-GFP treatment elicited delayed long-lasting antidepressant-like effects in the behavioral despair test, and this effect was blocked by the ablation of neurogenesis with X-irradiation. These results suggest that the involvement of hippocampal neurogenesis is required for the antidepressant-like behavioral effects of Ad-SIRT2.

Chronic unpredictable/variable/mild stress or chronic elevation of stress hormones have both been demonstrated to induce behavioral phenotypes mimicking those seen in major depression, which can be reversed by the administration of classic antidepressants^12^. In this study, we report that exposure to CUS can decrease expression of the SIRT2 protein in the hippocampus, and that the antidepressant fluoxetine can reverse the CUS-induced decrease in SIRT2. The reduced SIRT2 protein level in mice brain following CUS suggests that this protein plays a role in neuroprotection.

Previous studies have reported that HDAC inhibitor infusion into the nucleus accumbens after chronic stress produces robust antidepressant-like effects across several behavioral assays^13^. Rodent behavioral data demonstrate antidepressant-like effects of the class I HDAC inhibitor (sodium butyrate), as well as reduced psychostimulant-induced hyperactivity by sodium butyrate^14^. These findings support the utility of HDAC inhibitors as antidepressants. In contrast, our results demonstrate that tenovin-D3-treated rats showed increased immobility time, decreased percentage sucrose preference, spent less time in the open arms, and moved less in all areas of the elevated plus maze; also, CUS rats microinjected with the Ad-SIRT2-GFP displayed decreased immobility time, increased preference for sucrose solution, spent more time in the open arms, and moved more in all areas of the elevated plus maze, indicating that SIRT2 is involved in CUS-induced depression behavior, the inhibition of SRT2 in the hippocampus produces a depressive-like effect and that SIRT2 overexpression produces an antidepressant-like effect. Presumably, unlike class I and II HDACs, which require only zinc as a cofactor, SIRT2 depends on NAD^+^ for activity^15^. Therefore, more information is required regarding the identity and functions of these different classes of deacetylases.

The functional incorporation of adult-born neurons into hippocampal circuits increases hippocampal activity^16^. In this study, we found that tenovin-D3 significantly reduced the number of BrdU-positive cells, and that the number of apoptotic cells was increased in a tenovin-D3 dose-dependent manner. We also showed that DG transfected with Ad-SIRT2-GFP markedly increased BrdU incorporation, and that the apoptosis rate was decreased in the Ad-SIRT2-GFP-transfected cells. Previous reports stated that animals with higher hippocampal neurogenesis perform better in tests that are highly sensitive for hippocampal function in learning and memory^17^, while decreased hippocampal neurogenesis results in poorer performances^18^,^19^. Therefore, it is tempting to speculate that Ad-SIRT2-GFP-induced increases in hippocampal neurogenesis might contribute to its antidepressant-like behavioral effects.

Several studies have demonstrated that neurogenesis is required for some of the behavioral effects of the antidepressant drugs, and reduced neurogenesis affects hippocampal neuroplasticity in rodent models, thereby leading to anxiety, depression and related mood disorders, as well as cognitive disorders^20^. Therefore, studies have been based on strategies aimed at reducing neurogenesis in mice, such as focal hippocampal irradiation^21^,^22^,^23^. In this study, our results show that Ad-SIRT2-GFP-reduced immobility time was reversed in X-irradiation-treated rats, and X-irradiation abolished the effect of Ad-SIRT2-increased swimming time. Ad-SIRT2-GFP rats exposed to the sham procedure showed a significant decrease in immobility time and increase in swimming time at the end of 14-days of Ad-SIRT2-GFP treatment. These data suggest that Ad-SIRT2-GFP produces rapid and delayed antidepressant-like behavioral effects, and these effects are likely mediated, at least in part, by neurogenesis-dependent mechanisms.

In conclusion, this report demonstrates that hippocampal SIRT2 may be involved in the molecular mechanism for the regulation of depressive behaviors. Therefore, SIRT2 may play an important role in neurological disorders, and may represent a novel therapeutic target in the prevention of depression.

## Methods

### Animals

Male Sprague Dawley rats, weighing 250–300 g, were obtained from the Laboratory Animal Center of the Fourth Military Medical University. Rats were housed in groups of three. All animals were maintained on a 12 h light/dark cycle and received food and water *ad libitum*. Animals were habituated to housing conditions for 7 days prior to the beginning of experimental procedures.

### Chronic Unpredictable Stress

The chronic unpredictable stress (CUS) procedure was carried out in rats using a previously reported method^24^. Rats were randomly assigned to weight-matched control (n = 24) and chronic stress groups (n = 24). In brief, over the course of 3 weeks, rats were exposed daily to various stressors that were applied at different times of the day and included bright light, elevated platforms, predator odor, exposure to electric shocks, exposure to the context reminder of shocks, acoustic stimulation, forced swimming in tanks of different sizes, exposure to a novel arena, and exposure to elevated plus maze. Twenty-one days after the beginning of the CUS procedure, CUS rats were further divided into two groups. One group received intraperitoneal injection of fluoxetine (1 mg/kg) (n = 5) and the other received vehicle (saline) injection (n = 5), given 30 min before the application of stress daily. During the 28 days of fluoxetine or vehicle treatment, CUS rats were continuously exposed to stressors. All of the experimental procedures involving animals were conducted in accordance with the Institutional Animal Care Guidelines and approved ethically by the Administration Committee of Experimental Animals, Tangdu Hospital. All surgery was performed under sodium pentobarbital anesthesia (Sigma, St. Louis, MO), and all efforts were made to minimize suffering.

### Sucrose preference test

Sucrose preference was assessed based on a previously described methodology^25^. In brief, a subset of rats was tested for sucrose preference on day 0 and day 14 of the chronic unpredictable stress protocol. Rats were given 12 h (07:00–19:00) to habituate to a free choice between two bottles, both containing tap water. At 19:00, one bottle was replaced with 3% sucrose. The other bottle remained as tap water. Animals were given free choice between sucrose and tap water for 12 h (19:00–07:00). To prevent any possible effects of side preference, the position of the sucrose bottle was randomly distributed among the cages. Sucrose preference for each rat was defined as the average percentage of sucrose consumption of the total liquid consumption.

### Home-cage locomotion

Monitoring of locomotion was performed in the home cage using a computerized Inframot system, as described previously, which is based on infrared sensors located above the home cage of each rat. Mobility during the dark period (over 12 h per night) was measured for 5 days. Increasing the measurement time in untouched animals in their home cages increases the stability and reproducibility of locomotion data and therefore enhances the probability of observing significant and replicable behavioral changes.

### Exploration and novelty-induced behavior

Rats were placed in a 40 × 40 cm exploration box. Then, distance traveled and number of rearings were recorded automatically over 10 min. The distance traveled was estimated by beam breaks of 32 photo beam pairs, separated by 2.5 cm and placed 3 cm above the floor. The rearing activities of rats were detected by the number of beam breaks of 16 photo beam pairs, separated by 2.5 cm and placed 15 cm above the floor. The exploration box was thoroughly cleaned between tests.

### Forced swim test

The forced swim test was conducted in a cylindrical tank. The water temperature was kept at 24°C and the water level was such that the rat could not touch the bottom with its hind paws. Rats were individually placed in the cylinder for 15 min and returned to a home cage after drying quickly with a towel. On the following day, the subject rat was placed in the cylinder for 6 min. Animal behavior during the last 5 min was scored as described previously. Immobility time, which was defined as the total time during which animals remained floating with all limbs motionless, was counted for the last 5 min.

### Elevated plus maze

Rats were placed on the central platform facing a closed arm and allowed to explore the maze for 5 min. The total distance moved, time spent, and number of entries to the open, center, and closed arms were analyzed. Differences in the proportion of time spent in the open arms and close arms are indicative of differences in anxiety-like behavior.

### X-ray irradiation

X-ray irradiation was performed using a modified protocol from previously reported studies^26^,^27^. Rats were anesthetized and subjected to cranial irradiation using a Faxitron X-ray system. A lead shield was designed to protect the entire body of the rat from X-ray exposure while leaving a small oval-shaped area over the hippocampus open to X-ray exposure. On two consecutive days, 10 Gy each day was delivered over 15 min to anesthetized animals placed in a plastic restrainer. Sham controls were taken to the X-ray facility and anesthetized, but not subjected to irradiation. After a 4-week recovery period, SIRT2 (1 mg/kg) or saline was administered to the rats i.p. once per day for 14 consecutive days.

### Hippocampal neuron culture

Primary cultured hippocampal neurons were prepared and cultured as previously described^28^. Briefly, hippocampal neurons were prepared from rats and grown in Neurobasal media with B-27 supplement containing retinyl acetate, 0.5 mM Glutamax™-1. For initial plating, 25 μM L-glutamic acid was added. The media was replaced every 3 days. 10 μM of 5-Fluoro-5′-deoxyuridine was added to inhibit the growth of glial cells in the hippocampal neuron culture.

### Adenovirus production and infection *in vivo*

Carboxy-terminal FLAG-tagged SIRT2 cDNA was excised by PmeI and sub-cloned into a blunt-ended EcoRI site in a retro-viral vector pQCXIP. As a control vector, turbo-GFP cDNA was PCR-amplified from pLKO1-turboGFP and sub-cloned into a NotI site in pQCXIP. To produce retrovirus particles, sequence-verified plasmids were transfected into 293T cells using polyethylenimine with the helper vectors pCL10A1.

Ad-SIRT2-GFP and Ad-GFP were transfected into mouse hippocampus by stereotaxic microinjection at 1 μL per dentate gyrus. Five days later, mice were perfused transcardially, and serial brain sections were made. Effects of transfection *in vivo* could be observed by fluorescence microscopy.

### Microinjection and infusion of tenovin-D3

Rats were anesthetized with ketamine and placed in a stereotaxic apparatus. Tenovin-D3 was dissolved in a solution (physiological saline/polyethylene glycol 400, 4:1). The tenovin-D3 solution in 2 μL volume was microinjected into the DG (0.2 μL/min) at coordinates 2.3 mm posterior to the bregma, 1.3 mm lateral to the midline, and 2.0 mm below the dura. For the osmotic pump infusion, a 7 or 28 day Alzet osmotic mini-pump containing tenovin-D3 (6.7 μg/50 μL for 7 d or 26.8 μg/200 μL for 28 d) was placed subcutaneously in the back of the animals, and a brain infusion cannula connected to the pump was positioned at the above coordinates. The infusion rate was 0.25 μL/h. We anesthetized mice with ketamine and removed the osmotic pump after infusion tenovin-D3 solution treatment for 7 or 18 d.

### RT-PCR

Total RNA was isolated from frozen hippocampi using the miRNeasy kit. RNA samples were retro-transcribed using the First-strand cDNA synthesis kit. cDNA was diluted 1:10 and submitted to RT-PCR with the 7900HT Fast real-time PCR system using power SYBR Green PCR Master mix. The following primers were used for RT-PCR: SIRT2 (forward: GAACGCTGTCGCAGAGTCATC, reverse: GGTTGGCTTGAACTGCCCAG), and GAPDH (forward: AGCCACATCGCTCAGACAC, reverse: GCCCAATACGACCAAATCC). Quantification was performed by the relative standard curve method against the control GAPDH.

### Western blot

Hippocampal tissue was collected under both basal conditions and 30 min after exposure to stress. Each mouse hippocampus was homogenized in ice-cold homogenization buffer (10 mM HEPES/1.0 mM EDTA/2.0 mM EGTA/0.5 mM DTT/0.1 mM PMSF/1% NP-40) containing a protease and phosphatase inhibitor cocktail. Protein content in whole hippocampal samples was quantified using the protein assay. Proteins were resolved on 10% polyacrylamide gels, and transferred to nitrocellulose membranes. The membrane was then blocked in 5% non-fat milk and anti-SIRT2 was applied in this blocker overnight at 4°C. Peroxidase-labeled secondary IgG was applied for 1 h at room temperature followed by washing and bands were visualized using a chemiluminescence peroxidase substrate. The relative levels of each protein to the β-actin were analyzed.

### Neural cells proliferation assay

The proliferation of neural cells was determined by the BrdU incorporation assay. The same rats used for behavior testing were injected with BrdU 2 h after the last tenovin-D3 or Ad-SIRT2-GFP administration. Cell proliferation was confirmed by quantifying cell numbers using a cell counter (Kaihong, Beijng, China), and BrdU incorporation into DNA was quantified by using the Cell Proliferation ELISA BrdU kit (Takara, Dalian, China). To track the fate of BrdU-labeled cells, animals were sacrificed 28 d after the BrdU injection.

### Statistical analysis

Results were expressed as mean ± SD. Statistical analyses were performed with one-way ANOVA on behavioral tests, cell proliferation, and two-way ANOVA on the X-irradiation study. Student Newman Keuls or Tukey/Kramer *post hoc* comparisons followed ANOVAs.

## Author Contributions

K.J. and R.L. designed research; Z.L. and Q.Z. performed research; Y.D. analyzed data and prepared figures; R.L. and W.D. paiticipated in manuscript preparation and revisions. All authors edited and approved the final manuscript.
